# Mesenchymal Stromal Cell Derived Membrane Particles Are Internalized by Macrophages and Endothelial Cells Through Receptor-Mediated Endocytosis and Phagocytosis

**DOI:** 10.3389/fimmu.2021.651109

**Published:** 2021-03-15

**Authors:** Fabiany da Costa Gonçalves, Sander S. Korevaar, Maitane Ortiz Virumbrales, Carla C. Baan, Marlies E. J. Reinders, Ana Merino, Eleuterio Lombardo, Martin J. Hoogduijn

**Affiliations:** ^1^Nephrology and Transplantation, Internal Medicine, Erasmus Medical Center Transplantation Institute, Erasmus Medical Center, Rotterdam, Netherlands; ^2^Takeda Madrid, Cell Therapy Technology Center, Madrid, Spain

**Keywords:** mesenchymal stromal cells, membrane particles, extracellular vesicles, endocytosis, phagocytosis, macrophages, endothelial cells, immunomodulation

## Abstract

Mesenchymal stromal cells (MSC) are a promising therapy for inflammatory diseases. However, MSC are large and become trapped in the lungs after intravenous infusion, where they have a short survival time. To steer MSC immunoregulatory therapy beyond the lungs, we generated nm-sized particles from MSC membranes (membrane particles, MP), which have immunomodulatory properties, and investigated their internalization and mode of interaction in macrophages subtypes and human umbilical vein endothelial cells (HUVEC) under control and inflammatory conditions. We found that macrophages and HUVEC take up MP in a dose, time, and temperature-dependent manner. Specific inhibitors for endocytotic pathways revealed that MP internalization depends on heparan sulfate proteoglycan-, dynamin-, and clathrin-mediated endocytosis but does not involve caveolin-mediated endocytosis. MP uptake also involved the actin cytoskeleton and phosphoinositide 3-kinase, which are implicated in macropinocytosis and phagocytosis. Anti-inflammatory M2 macrophages take up more MP than pro-inflammatory M1 macrophages. In contrast, inflammatory conditions did not affect the MP uptake by HUVEC. Moreover, MP induced both anti- and pro-inflammatory responses in macrophages and HUVEC by affecting gene expression and cell surface proteins. Our findings on the mechanisms of uptake of MP under different conditions help the development of target-cell specific MP therapy to modulate immune responses.

## Introduction

Mesenchymal stromal cells (MSC) are self-renewing cells found in several postnatal organs and tissues from which they can be easily isolated and expanded in *in vitro* conditions (1, 2). Their immunomodulatory and regenerative properties enable MSC to be used as an potential therapy for several diseases, including inflammatory bowel diseases (3, 4), rheumatoid arthritis (5), atherosclerosis (6), and kidney injury (7). However, culture-expanded MSC are large and become trapped in the pulmonary vascular network after intravenous administration (8–11). MSC are no longer detected in the lungs after 24 h and their cellular debris is phagocytized and distributed to other sites of the body (12). Moreover, MSC transplantation can lead to practical complications resulting from the use of living cells, including immune responses, thrombosis, tumor formation, and transmission of infections (2, 13–15).

To steer MSC therapy beyond the lungs, we generated nm-sized vesicles from MSC membranes (membrane particles, MP). MP have a spherical shape and are composed of MSC outer cell membranes and organelles (unpublished data). Because of their small size and vesicle shape, MP are potentially capable of overcoming the pulmonary barrier. These particles contain the membrane-bound proteins of MSC, several of which have immunomodulatory, metabolic, and adhesion functions. We previously reported that MP possess similar immune regulatory properties as MSC with respect to the modulation of monocyte function after being taken up by these cells (16). We also found that MP, like naturally occurring extracellular vesicles, are efficiently taken up by endothelial cells and modulate their function (unpublished data), and (17, 18). However, the specificity and mechanisms of MP uptake by target cells remain unclear. Thorough understanding of the mechanisms of MP uptake by different cell types is of great importance for the use of MP for immune and regenerative therapy.

The mechanisms of particle uptake involve protein interactions that facilitate subsequent endocytosis. The internalization process can be divided into receptor-mediated endocytosis, phagocytosis/macropinocytosis, and passive penetration (19, 20). Endocytosis is mediated by specific cell surface receptors. These are transmembrane proteins that interact with specific extracellular molecules on vesicles and subsequently initiate endocytosis, resulting in heparan sulfate proteoglycans (HSPG)-, dynamin-, clathrin-, and caveolin-mediated endocytosis (21, 22). Phagocytosis and macropinocytosis are mediated by the polymerization of actin and phosphoinositide 3-kinases (PI3K), which allow the insertion of the cell membrane in the formation of phagosomes (23–25). Moreover, the properties of particles in combination with characteristics of the cellular and extracellular environments, such as temperature, exposure time, inflammatory environment, and type of receptor cells, can govern the localization of particles in the target cells (19, 24).

The ability of MP to interact with host cells, deliver their biological effect, and provoke an immunological and regenerative response is dependent on their uptake. Understanding the mechanisms of uptake allows steering and conditioning of their uptake and thereby control of their potential therapeutic effects. Here, we characterized human MP uptake and internalization by macrophages subtypes and endothelial cells, which are among the first cell types to be exposed to infused MP and play a crucial role in immune responses, and examined their function under quiescent and inflammatory conditions.

## Materials and Methods

### Isolation and Culture of MSC

MSC were obtained from subcutaneous adipose tissue from 13 healthy human donors that became available during the living kidney donation procedure. All donors provided written informed consent as approved by the Medical Ethical Committee of the Erasmus University Medical Center Rotterdam (protocol no. MEC-2006-190). MSC were isolated and phenotypically characterized by the expression of CD13, CD73, CD90, and CD105 and the absence of CD31 and CD45 as described previously (16). MSC were cultured in minimum essential medium-α (MEM-α) (Sigma-Aldrich, St. Louis, MO, USA) supplemented with 100 IU/ml penicillin, 100 mg/ml streptomycin (P/S), 2 mM L-glutamine, and 15% fetal bovine serum (FBS) (all Lonza, Verviers, Belgium). Cultures were kept at 37°C, 5% CO_2_, and 95% humidity. At 90% confluence, adherent cells were collected from culture flasks by incubation in 0.05% trypsin-EDTA (Life Technologies, Bleiswijk, The Netherlands) at 37°C. MSC between passages 2 and 6 were used for MP generation.

### Generation of MSC Membrane Particles

MSC were collected, counted, washed twice with phosphate-buffered saline (PBS), and centrifuged at 2,000 × g for 5 min. The MSC pellet was incubated in Milli-Q water at 4°C for ~20 min to induce osmotic lysis and release of cell nuclei. This step was carefully monitored by an optical microscope and stopped when nuclei were released from the cells. Cell extracts were isolated from unbroken cells and nuclei by centrifugation at 2,000 × g for 20 min. Then, the supernatant was transferred to Amicon Ultra-15 filter tubes (100 kDa pore size) and concentrated by centrifugation at 4,000 × g for 45 min. The concentrated pellet consisted of crude membranes and was diluted in filtered PBS. To prepare a small and uniform size of MP, the membranes were extruded three times through polycarbonate membrane filters (Merck, KGaA, Darmstadt, Germany) using LiposoFast LF-50 (AVESTIN Europe, Mannheim, Germany) at 20 psi, first with a pore size filter of 800 nm, then 400 nm, and finally 200 nm. All procedures were performed on ice. To obtain fluorescent MP, MSC were labeled with the red fluorescent PKH-26 dye (PKH-MP), which intercalates into lipid bilayers, according to the manufacturer's instructions (Sigma-Aldrich), before generation of MP.

### Nanoparticle Tracking Analysis

Absolute size distribution and concentration of MP was performed using the NTA by NanoSight NS300 (NanoSight Ltd.). NTA automatically tracked and sized particles based on Brownian motion and the diffusion coefficient. First, the samples were diluted to obtain the right number of particles (1 × 10^8^ particles/ml) in accordance with the manufacturer's recommendations. Three measurements per sample (30 s/measurement) were captured under the following conditions: temperature 23.61 ± 0.8°C; viscosity 0.92 ± 0.02 cP, frames per second (25). After capture, the videos were analyzed to give the mean, mode, median, and estimated concentration for each particle size with a detection threshold 3 (determined with a protein solution).

### Cryo-Transmission Electron Microscopy

The morphology of MP was visualized by Cryo-TEM. A thin aqueous film was foMPed by applying a 3 μl droplet of MP suspension to a specimen bare EM grid. For that, glow-discharged holey carbon grids were used. Then, the grid was blotted against filter paper, leaving a thin sample film covering the grid holes. These films were vitrified by immersing the grid into ethane, which was maintained at its melting point by liquid nitrogen using a Vitrobot (Thermo Fisher Scientific Company, Eindhoven, Netherlands) to prevent samples from freezing at 95% humidity. The vitreous sample films were transferred to a Tecnai Arctica microscope (Thermo Fisher Scientific, Eindhoven, Netherlands). Images were taken at 200 Kv with a field emission gun using a direct electron detector Falcon III (Thermo Fisher Scientific).

### Culture of Human Monocytic Cell Line THP-1

THP-1 (ATCC: TIB-202) is a human monocytic cell line derived from an acute monocytic leukemia patient. THP-1 cells were cultured in RPMI 1640-GlutaMAX (Gibco, Thermo Fisher Scientific) supplemented with 10% heat inactivated FBS and 1% P/S at 37°C under 5% CO_2_. Cells were grown to a density of 1–8 × 10^5^ cells/ml and used for experiments between passage 2 and 10. Differentiation of THP1 cells into macrophage-like cells was induced by stimulation with 50 ng/ml phorbol-12-myristate-13-acetate (PMA) (Sigma) for 72 h. Following differentiation, PMA-containing media was replaced with fresh media, and cells were rested in culture for 24 h.

### Culture of Human Umbilical Vein Endothelial Cells

HUVEC pooled from multiple donors were purchased from Promocell (Promocell, Germany). Cells were cultured in endothelial cell basal medium (EBM, Cambrex Bio Science Walkersville, Inc., Walkersville, MD, USA), endothelial cell growth medium supplements (EGM, Cambrex Bio Science), 5% FBS and 1% P/S at 37°C under 5% CO_2_. At 90% confluence, HUVEC were collected by incubation in 0.05% trypsin-EDTA at 37°C. HUVEC between passages 2 and 7 were used for the experiments.

### Uptake of MP by Macrophages and HUVEC

THP-1 macrophages (2 × 10^4^ cells/ml) and HUVEC (1 × 10^4^ cells/ml) were cultured with PKH-MP at different ratios (Cell: MP; 1:10,000, 1:50,000, 1:100,000) at 37°C, 5% CO_2_, and 95% humidity. To determine if the uptake of MP was *via* an active process, cells were alternatively incubated at 4°C. MP uptake by macrophages and HUVEC was analyzed through detection of PKH positive cells by flow cytometry (FACS Canto II, Becton Dickinson) at 1, 6, and 24 h.

For confocal microscopy analysis, THP-1 macrophages, and HUVEC were cultured with PKH-MP (ratio 1:50,000) for 24 h. Cell membranes of macrophages and HUVEC were labeled with CD81-APC (BioLegend, San Diego, CA) and the nuclei with 10 μM Hoechst 33342. Images were performed on a Leica TCS SP5 confocal microscope (Leica Microsystems B.V., Science Park Eindhoven, Netherlands) equipped with Leica Application Suite – Advanced Fluorescence (LAS AF) software, DPSS 561 nm lasers, using a 60 × (1.4 NA oil) objective. Images were processed using ImageJ 1.48 (National Institutes of Health, Washington, USA).

### MP Uptake Inhibitors

Cells were preincubated for 30 min at 37°C in complete medium containing Heparin (0.1–100 μg/ml; H3149; Sigma), Dynasore (20–160 μM; D7693; Sigma), Chlorpromazine (1–50 μM; C8138; Sigma), Nystatin (5–40 μg/ml; N6261; Sigma), Cytochalasin D (0·25–2 μM; C8273; Sigma), or Wortmannin (0.1–10 μM; W1628; Sigma). Cell viability was measured by flow cytometer using BD Via-Probe™ Cell Viability Solution containing 7-AAD. PKH-MP were then added to the cells (ratio 1:100,000) and analyzed after 6 h by flow cytometer. Following the results of these experiments, combination treatment with inhibitors of endocytosis (10 μg/ml Heparin and 80 μM Dynasore) and phagocytosis (5 μM Wortmannin) was also tested. Drug vehicle controls were used for the experiments: 0.1% PBS as a control for Heparin, Chlorpromazine, Nystatin, 0.1% dimethyl sulfoxide (DMSO) for Dynasore, Cytochalasin D, and Wortmannin.

### MP Uptake by Macrophages and HUVEC Under Inflammatory Conditions

For simulating inflammatory conditions *in vitro*, macrophages were primed with fresh medium supplemented with 20 ng/ml IFNγ (Gibco, Thermo Fisher Scientific) and/or 100 ng/ml lipopolysaccharides (LPS) (Sigma) to generate pro-inflammatory M1 macrophages. For anti-inflammatory conditions, macrophages were primed with 20 ng/ml IL-4 (PeproTech, London, UK) and/or 20 ng/ml IL-13 (PeproTech) to generate anti-inflammatory M2 macrophages. The incubation time was 24 h for all the conditions. PKH-MP were then added to macrophages (ratio 1:50,000) and incubated for another 24 h after which uptake was analyzed by flow cytometer and confocal microscopy.

HUVEC were primed with fresh medium supplemented with 10 ng/ml TNFα and/or 50 ng/ml IFNγ for 24 h. MP were added to the cells (ratio 1:50,000) and incubated for another 24 h after which uptake was analyzed by flow cytometer. After 48 h, HUVEC were collected for functional analysis by flow cytometer, after staining with HLA-I-PacBlue (BD Biosciences, San Jose, CA), HLA-II-PerCP (BioLegend), CD40-APC (Miltenyi Biotec, Bergisch Gladbach, Germany), CD144-PE (Ebioscience, Thermo Fisher Scientific).

### Quantitative RT-PCR Analyses

Macrophages were polarized in M1 and M2 macrophages by LPS, IFNγ, IL-4, and IL-13, respectively. HUVEC were primed with TNFα and IFNγ. After 24 h, MP were added to macrophages or HUVEC (ratio 1:50,000) and incubated for 48 h. Cells were harvested, washed with PBS-diethylpyrocarbonate (DEPC; Sigma-Aldrich), and stored at −80°C. Total RNA was isolated and 500 ng used for complementary DNA (cDNA) synthesis. Gene expression was determined by Quantitative Real-Time PCR (qPCR) using the TaqMan Universal PCR Master Mix (Life Technologies), and the assay-on-demand primer/probes for CXCL10 (Hs00171042), CCR7 (Hs00171054), Interleukine-1β (IL-1β, Hs00174097), Interleukine-10 (IL10, Hs00174086), tumor necrosis factor-α (TNFα, Hs99999043), transforming growth factor- β (TGFβ, Hs00171257), CCL22 (Hs00171080), and CD209 (Hs01588349) for macrophages; and Interleukine-6 (IL-6, Hs00174131), Interleukine-8 (IL-8, Hs00174114), and Endothelin (Hs 00174961) for HUVEC. 18S (Hs99999901) and glyceraldehyde 3-phosphate dehydrogenase (GAPDH, Hs99999905) mRNA served as a housekeeping genes for macrophages and HUVEC normalization, respectively.

### Statistical Analysis

Data were analyzed for statistical significance by Student's *t*-test and one-way and two-ways ANOVA, and analysis using GraphPad Prism 5 software. *P* < 0.05 was considered significant.

## Results

### Characterization of MP

**Figure 1:**
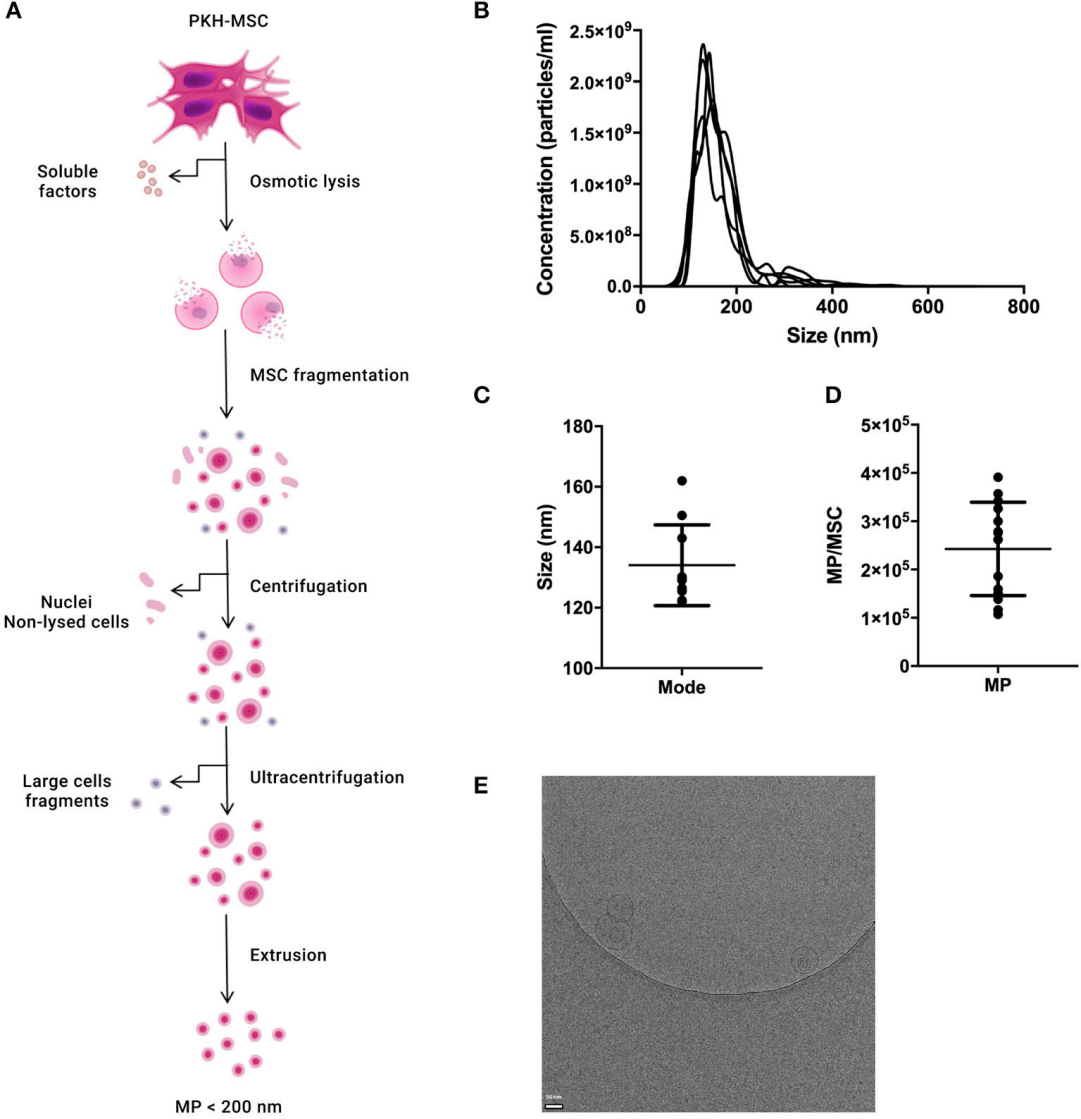
Characterization of reconstructed membranes (MP) generated from MSC. **(A)** Schematic overview of the generation of MP. **(B)** Nanoparticle tracking analysis (NTA) profile of MP. **(C)** MP size distribution. **(D)** Number of MP generated per MSC. **(E)** Cryo-electron microscopy image of MP. MP (arrow) show a spherical shape and a visible lipid bilayer. Data in C and D are presented as mean ± SD from 12 independent preparations of MP.

MP were generated from culture-expanded MSC and characterized by NTA and Cryo-TEM to determine their concentration, size distribution, and morphology (Figure 1A). The percentage of particles with a size larger than 200 nm was lower than 5% (Figure 1B). The mode size of MP was 134.1 ± 13.3 nm (Figure 1C). The average number of MP generated per MSC was 2.4 × 10^5^ ± 10.0 × 10^4^ (Figure 1D) There was no significant difference in size distribution or concentration between MP and fluorescently-labeled MP (PKH-MP) (Supplementary Figure 1). Cryo-TEM showed that MP have a spherical shape and a discernible lipid bilayer. Some MP were encapsulated inside larger MP (Figure 1E).

### Macrophages and HUVEC Internalize MP in a Dose-, Time-, and Temperature-Dependent Manner

**Figure 2:**
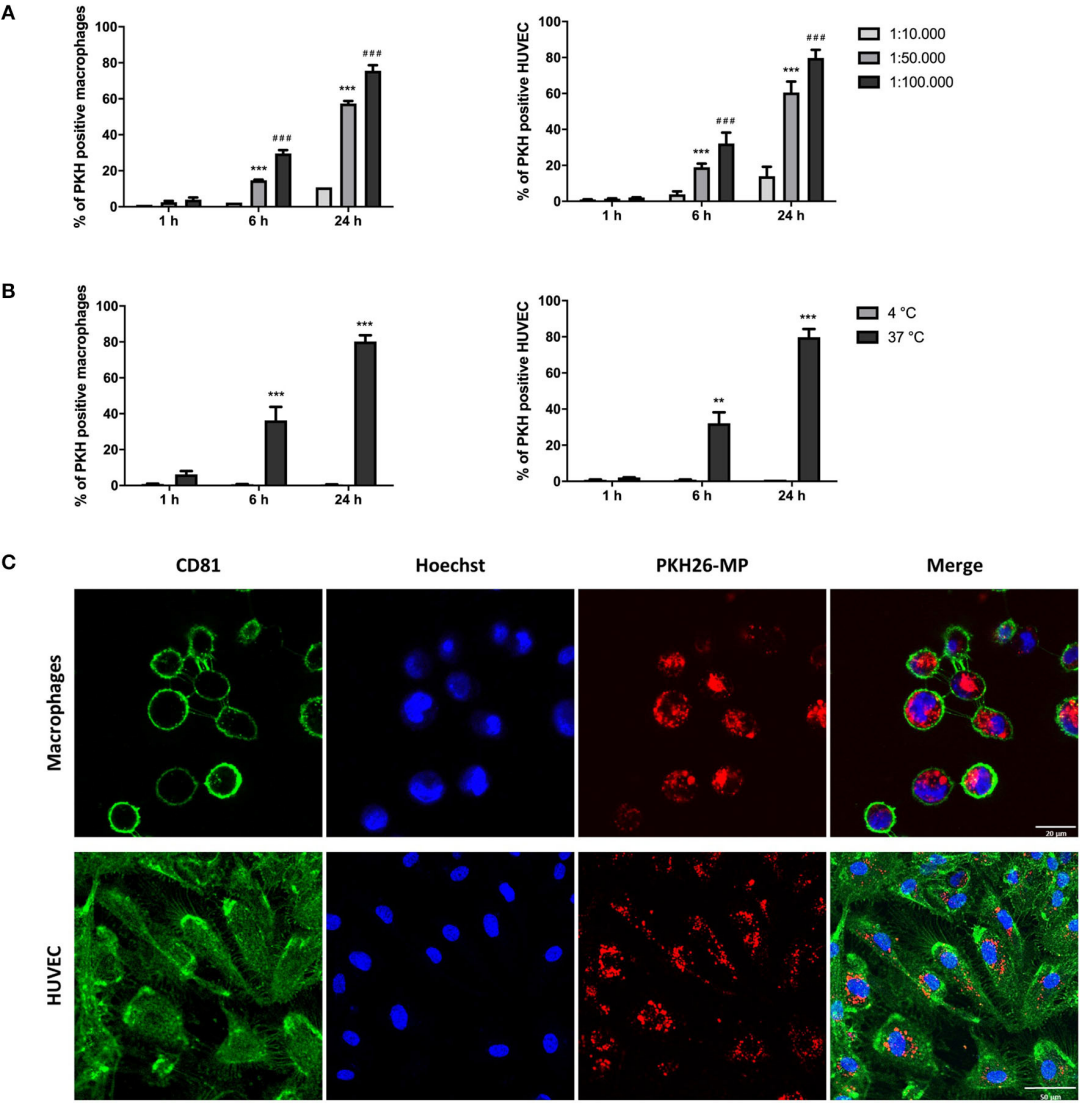
Macrophages and HUVEC internalize MP in a dose-, time-, and temperature-dependent manner. MSC were labeled with PKH-26 before the generation of MP (PKH-MP). PKH-MP were added to macrophages or HUVEC (ratios 1:10,000, 1:50,000, 1:100,000), incubated for 1, 6, and 24 h at 4 or 37°C, and analyzed by flow cytometer or confocal microscopy. **(A)** Percentage of macrophages and HUVEC positive for PKH-MP in different doses of MP over time, and **(B)** at 4 and 37°C over time. **(C)** Representative confocal microscopy analysis of MP uptake by macrophages and HUVEC (ratio 1:50,000) at time point 24 h. Staining for CD81 cell membrane (green), Hoechst 33,342 nucleus (blue), and PKH26-MP (red) demonstrated that MP are internalized by macrophages and HUVEC. Data are presented as mean ± SD from 3 to 4 experiments. ***P < *0.001 vs*. ratio 1:10,000 and ^*###*^*P* < *0.001 vs*. ratio 1:50,000 in **(A)**; **P < *0.001* and **P < *0.001 vs*. 4°C in **(B)**. Scale bars: 20 μm (macrophages) and 50 μm (HUVEC).

THP-1 macrophages and HUVEC were cultured with PKH-MP at different ratios (1:10,000, 1:50,000, 1:100,000) at 4 and 37°C and analyzed by flow cytometer after 1, 6, and 24 h. We found that macrophages and HUVEC uptake MP in a dose and time-dependent manner. MP uptake increased with rising concentration and time of incubation (Figure 2A) and was completely inhibited at 4°C, which indicates a temperature-dependent process (Figure 2B). The interaction of MP with macrophages and HUVEC was visualized by confocal immunofluorescence microscopy. The confocal microscopy images showed that both macrophages and HUVEC internalized PKH-MP (Figure 2C).

### MP Internalization Depends on HSPG-, Dynamin- and Clathrin-Mediated Endocytosis, but Does Not Involve Caveolin-Mediated Endocytosis

**Figure 3:**
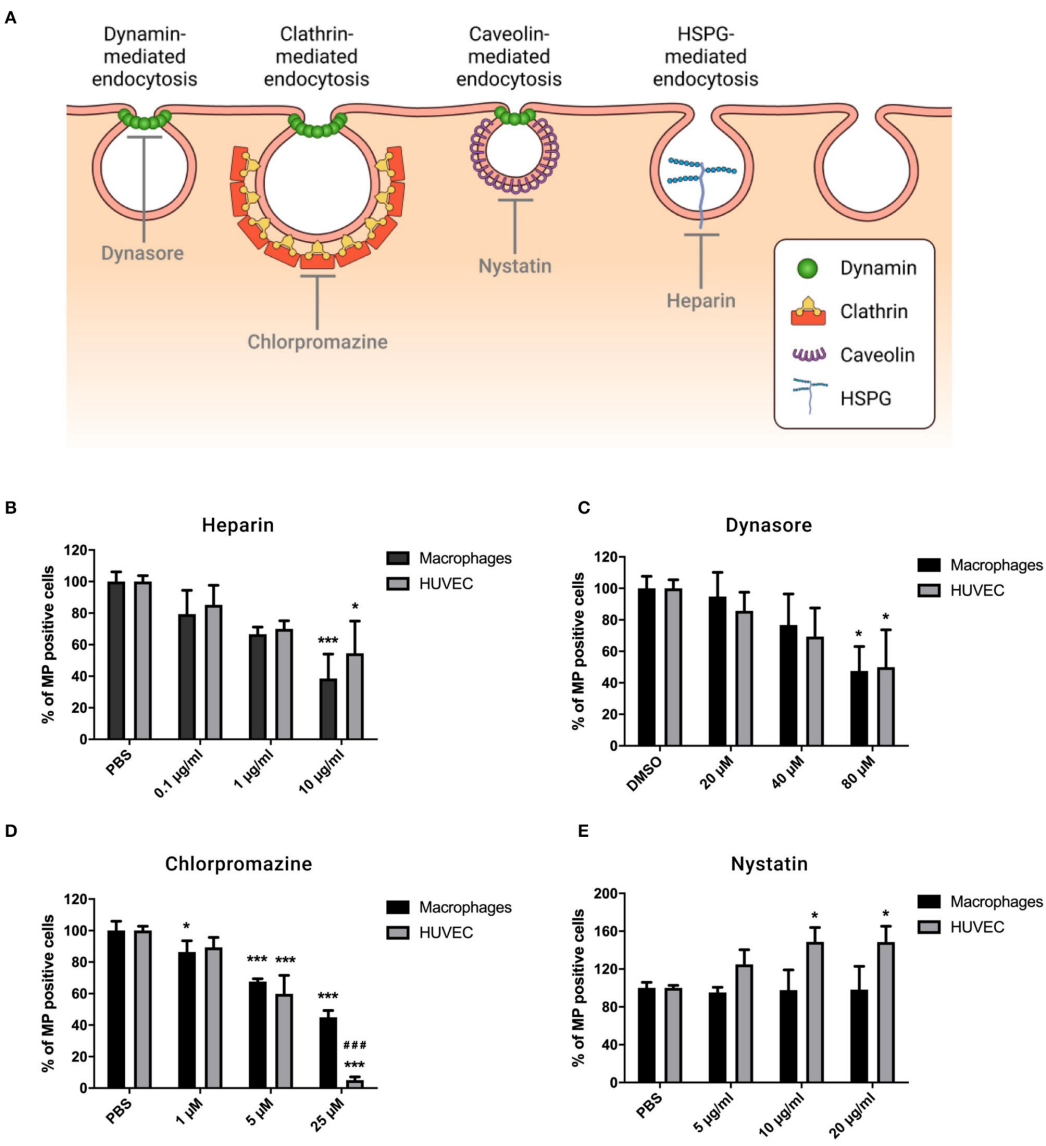
MP uptake by macrophages and HUVEC depends on HSPG-, dynamin-, and clathrin-mediated endocytosis. Cells were preincubated for 30 min at 37°C in complete medium containing Heparin (0.1, 1, and 10 μg/ml), Dynasore (20, 40, and 80 μM), Chlorpromazine (1, 5, and 25 μM), or Nystatin (5, 10, and 20 μg/ml). PKH-MP were then added to cells (ratio 1:100,000) and analyzed after 6 h by flow cytometer. PBS and 0.1% DMSO were used as a control. **(A)** Receptor-mediated endocytosis pathways. **(B)** Percentage of macrophages and HUVEC positive for PKH-MP in the presence of increasing concentrations of Heparin **(B)**, Dynasore **(C)**, Chlorpromazine **(D)**, and Nystatin **(E)**. Data are presented as mean ± SD from 4 to 6 experiments. *P < *0.05* and ***P < *0.001 vs*. vehicle; ^*###*^*P* < *0.001 vs*. macrophages.

To elucidate whether specific endocytic processes are responsible for MP internalization, macrophages, and HUVEC were pre-treated with pharmacological inhibitors that interfere in different endocytosis pathways as described in Figure 3A. First, the effect of different concentrations of the inhibitors on viability of the recipient cells was evaluated (Supplementary Figure 2). For the following experiments, we used the maximum doses that did not affect cell viability.

MP bind to cells through cell-surface receptors, after which they are internalized. Our results showed that heparan sulfate proteoglycans (HSPGs) are involved in binding of MP as the recipient cell-MP interaction is partially inhibited in the presence of heparin (a soluble analog of HSPGs) (21) in a dose-dependent manner (Figure 3B). Dynamin is a regulator of the endocytosis processes and is involved in clathrin- and caveolin-dependent routes (26). We blocked dynamin activity by its selective inhibitor Dynasore, which dose-dependently reduced cellular uptake of MP by macrophages and HUVEC (Figure 3C). To discriminate endocytotic routes, we used Chlorpromazine and Nystatin to block clathrin- and caveolin-mediated endocytosis, respectively. We observed that Chlorpromazine led to a dose-dependent inhibitory effect on MP uptake, and it was greater in HUVEC than in macrophages (Figure 3D). Nystatin had no significant effect on the uptake of MP by macrophages but it slightly increased uptake of MP by HUVEC (Figure 3E). No effect was observed with the carrier controls, PBS, or DMSO.

### MP Internalization Involves Phagocytosis and Macropinocytosis

**Figure 4:**
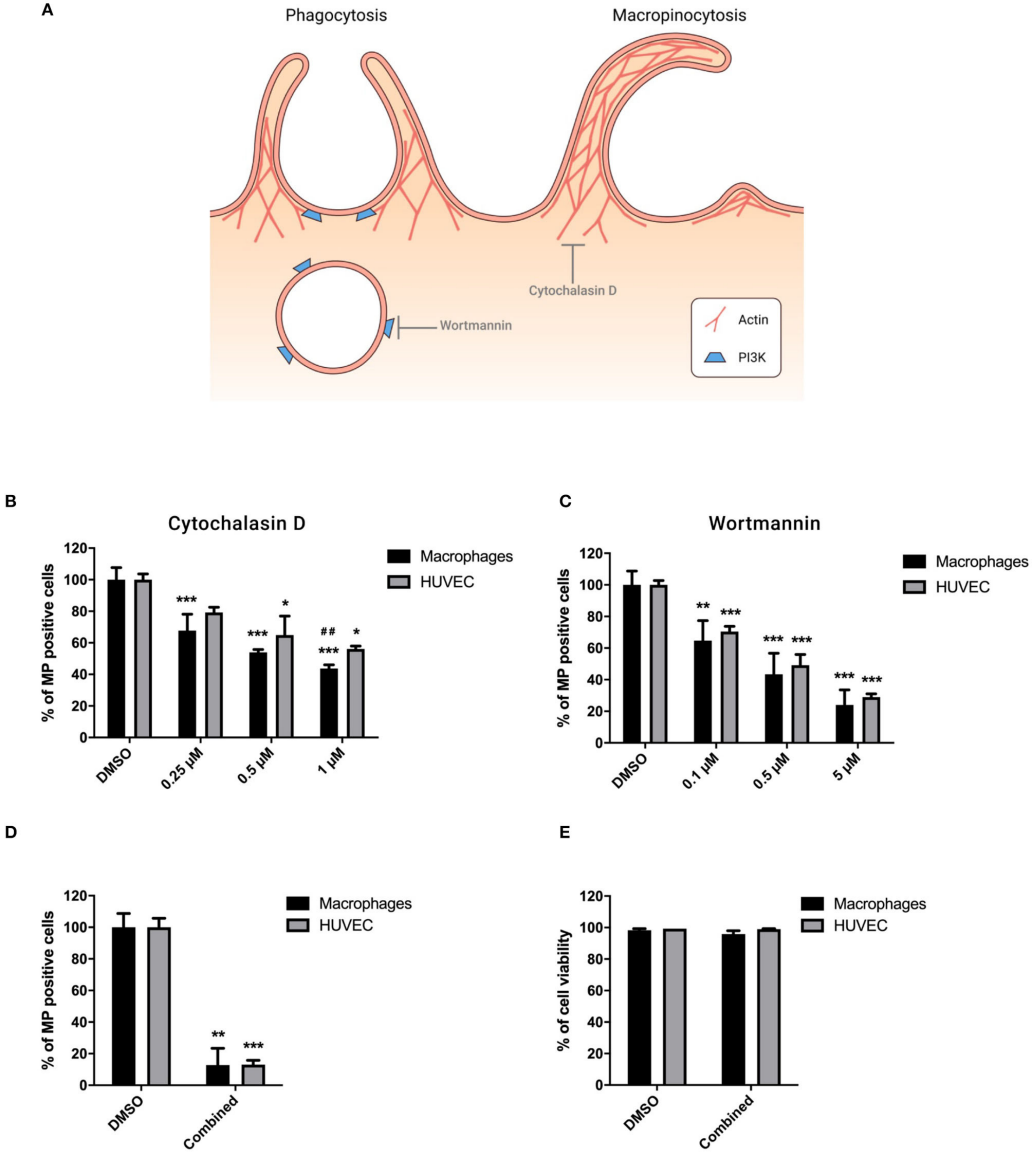
MP internalization involves phagocytosis and macropinocytosis. Cells were preincubated for 30 min at 37°C in complete medium containing Cytochalasin D (0.25, 0.5, and 1 μM), Wortmannin (0.1, 0.5, and 10 μM), or combination treatment with inhibitors of endocytosis (10 μg/ml Heparin and 80 μM Dynasore) and phagocytosis (5 μM Wortmannin). PKH-MP were then added to cells (ratio 1:100,000) and analyzed after 6 h by flow cytometer. PBS and 0.1% DMSO were used as a control. **(A)** Phagocytosis and macropinocytosis mechanisms. Percentage of macrophages and HUVEC positive for PKH-MP in the presence of increasing concentrations of Cytochalasin D **(B)**, Wortmannin **(C)**, and combined treatment **(D)**. **(E)** Percentage of macrophage and HUVEC viability after combined treatment. Data are presented as mean ± SD from 4 to 6 experiments. *P < *0.05*, **P < *0.01* and ***P < *0.001 vs*. vehicle; ^*##*^*P* < *0.001 vs*. HUVEC.

Phagocytosis and macropinocytosis involve the polymerization of actin and phagosome formation mediated by PI3K protein (23, 24) as described in Figure 4A. Cytochalasin D, an inhibitor of actin polymerization, dose-dependently inhibited MP uptake, and its effect was stronger in macrophages than HUVEC (Figure 4B). Wortmannin, an inhibitor of PI3K, led to a dose-dependent blocking of MP uptake by both cell types (Figure 4C). Combination treatment with 10 μg/ml Heparin, 80 μM Dynasore, and 5 μM Wortmannin to block MP binding, endocytosis, and phagocytosis resulted in a synergistic reduction of MP uptake of more than 85% in both HUVEC and macrophages (Figure 4D). No significant change in cell viability was observed following combined treatment (Figure 4E).

### MP Modulate Macrophage Function

To investigate the uptake of MP by macrophages under different immunological conditions, we primed macrophages with LPS and IFNγ (pro-inflammatory M1 macrophages) or IL-4 and IL-13 (anti-inflammatory M2 macrophages). Inflammatory conditions led to macrophage elongation and anti-inflammatory conditions resulted in more rounded and loosely attached macrophages within 24 h of stimulation (Supplementary Figure 3A). M1 macrophages produced higher gene expression levels of CXCL10, CCR7, IL-10, and TGFβ, whereas M2 macrophages produced higher levels of CCL22 and CD209 within 72 h of stimulation (Supplementary Figure 3B). Significant changes in gene expression were not observed for IL-10.

**Figure 5:**
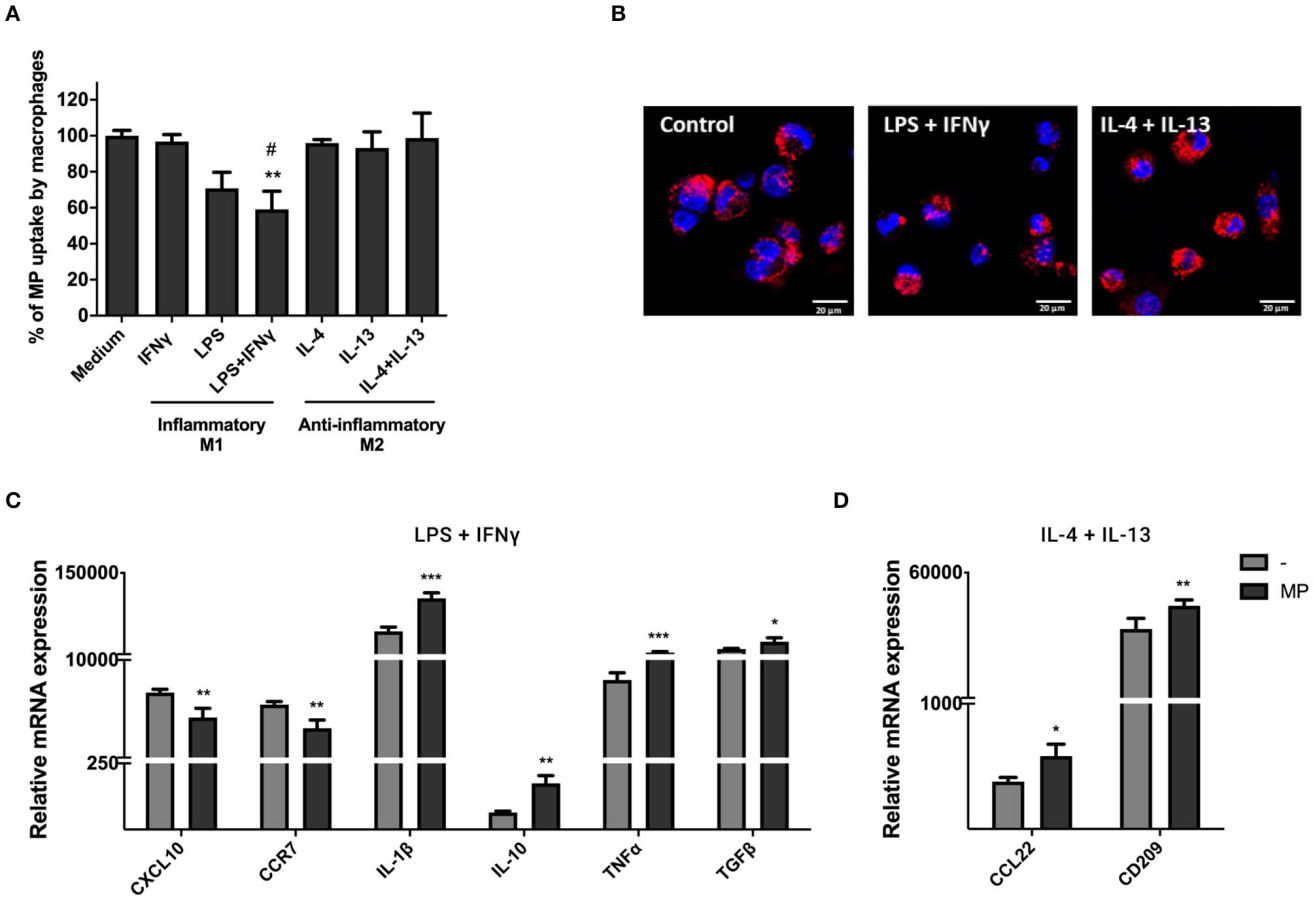
MP uptake under pro- and anti-inflammatory conditions modulates macrophages function. Macrophages were primed with 100 ng/ml LPS and 20 ng/ml IFNγ (M1 macrophages) or with 20 ng/ml IL-4 and 20 ng/ml IL-13 (M2 macrophages). **(A,B)** PKH-MP were added to macrophages (ratio 1:50,000) and incubated for 24 h for uptake analyze by flow cytometer and confocal microscopy. **(A)** Percentage of macrophages positive for PKH-MP in different culture conditions. **(B)** Representative confocal microscopy analysis of MP uptake by macrophages under stimulation of IFNγ + LPS or IL-4 + IL-13. **(C,D)** Macrophages were separated from MP and assessed by real-time RT-PCR after 48 h. mRNA expression of macrophages treated with MP in the presence of **(C)** LPS + IFNγ (CXCL10, CCR7, IL-1, IL-10, TNFα, and TGFβ) and **(D)** IL-4 + IL-13 (CCL22 and CD209). Data are presented as mean ± SD from 6 experiments. *P < *0.05*, **P < *0.01*, and ***P < *0.001 vs*. medium control or *vs*. macrophages without MP and ^#^*P* < *0.05* vs. macrophages stimulated with IL-4 + Il-13. Scale bar: 20 μm.

PKH-MP were added to macrophages and assessed by flow cytometry after 24 h. We found that anti-inflammatory M2 macrophages take up more efficiently MP than pro-inflammatory M1 macrophages, which significantly decreased their ability to internalize MP as indicated in Figure 5A. Confocal images showed that M1 and M2 macrophages internalized PKH-MP into the cytoplasm (Figure 5B). mRNA expression of a number of genes with pro- and anti-inflammatory function was analyzed in macrophages by qPCR after 48 h of stimulation with MP to determine whether MP could affect macrophages gene expression and immune function. MP induced anti- and pro-inflammatory genes in macrophages exposed to LPS and IFNγ treatment. MP decreased the gene expression of pro-inflammatory chemokines CXCL10 and CCR7 and increased anti-inflammatory cytokine IL-10 and TGFβ in M1 macrophages. However, MP also increased the gene expression of pro-inflammatory cytokines IL-1β and TNFα (Figure 5C). Following IL-4 and IL-13 treatment, MP increased gene expression of the anti-inflammatory chemokine CCL22 and the immunoregulatory marker CD209 in M2 macrophages (Figure 5D).

### MP Uptake Modulates HUVEC Function Under Inflammatory Conditions

To investigate the uptake of MP by HUVEC in inflammatory conditions, we primed HUVEC with single or combined TNFα and IFNγ doses. HUVEC exposed to TNFα and IFNγ alone led to cell elongation 24 h after stimulation and this morphological change was more pronounced when TNFα and IFNγ were combined (Supplementary Figure 4A). Moreover, HUVEC-surface expression level of HLA-I, HLA-II, and costimulatory molecule CD40 increased after single γ or combined doses of TNFα and IFNγ for 72 h (Supplementary Figure 4B). TNFα alone also increased HLA-I level. Significant changes on HUVEC-surface expression were not observed for CD144 (vascular endothelial cadherin), which is involved in the formation of endothelial intercellular junctions. TNFα and IFNγ upregulated the mRNA expression of pro-inflammatory cytokines IL-6 and IL-8 and of the angiogenic mediator endothelin in HUVEC after 72 h (Supplementary Figure 4C). TNFα alone also increased IL-8 gene expression.

**Figure 6:**
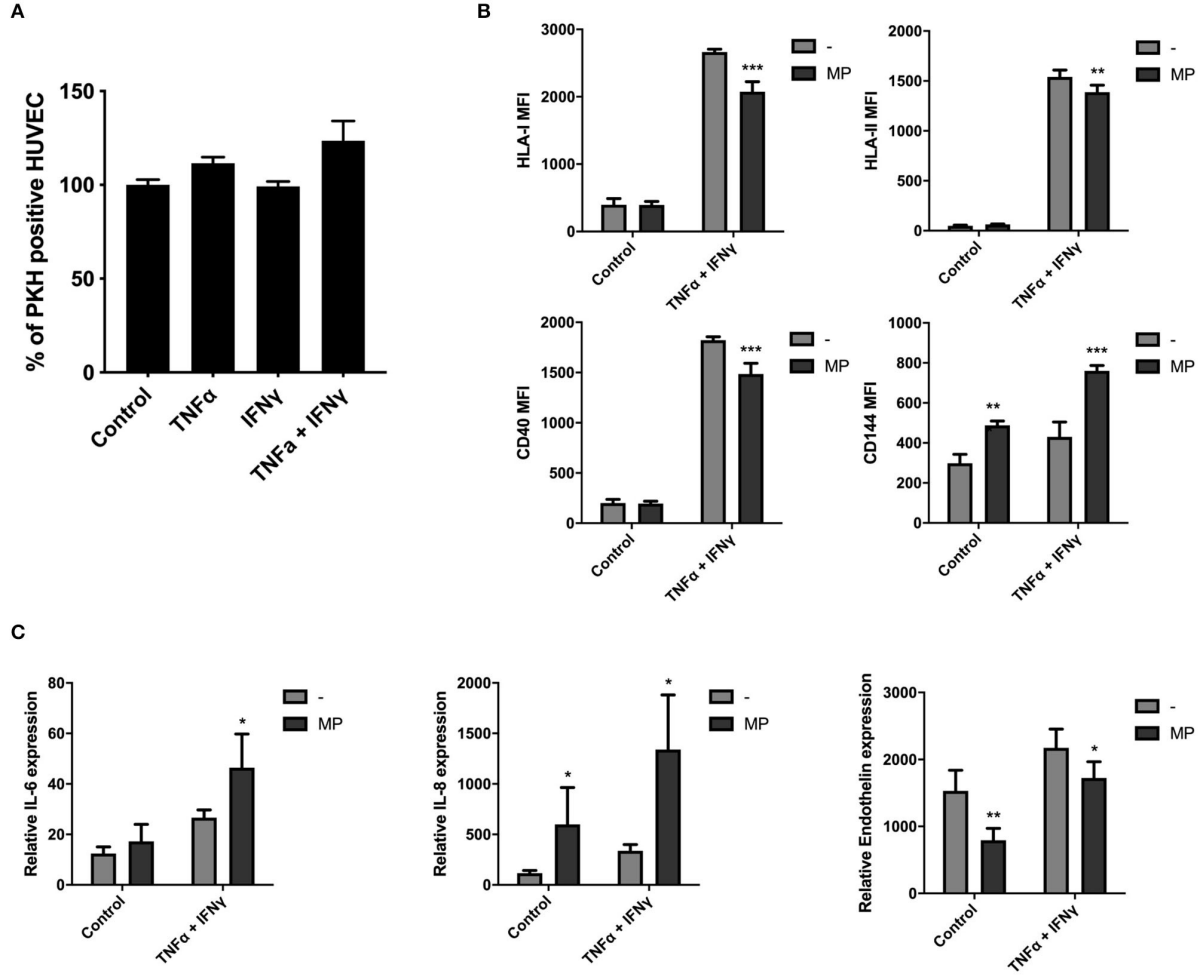
MP uptake modulates HUVEC function under inflammatory conditions. HUVEC were primed with single or combined doses of 10 ng/ml TNFα and 50 ng/ml IFNγ. PKH-MP were added to the cells (ratio 1:50,000) and incubated for 24 h after which uptake was analyzed by flow cytometer. **(A)** Percentage of HUVEC positive for PKH-MP in different culture conditions. **(B,C)** HUVEC were separated from MP and assessed by flow cytometer and real-time RT-PCR after 48 h. **(B)** HUVEC surface levels of HLA-I, HLA-II, CD40, and CD144 and **(C)** mRNA expression of HUVEC for IL-6, IL-8, and endothelin after treated with MP under quiescent or TNFα + IFNγ conditions. Data are presented as mean ± SD from 6 experiments. *P < *0.05*, **P < *0.01*, and ***P < *0.001 vs*. medium control or HUVEC without MP.

We found that MP uptake by HUVEC after 24 h was not affected under TNFα and IFNγ stimulation, alone or in combination as indicated in Figure 6A. HUVEC were collected for functional analysis by flow cytometry and qPCR after 48 h of stimulation with MP to determine whether MP affected HUVEC protein surface and gene expression levels in quiescent and inflammatory environment (TNFα and IFNγ). MP decreased the surface expression level of HLA-I, HLA-II, and CD40 costimulatory molecule on HUVEC under inflammatory conditions (Figure 6B). Moreover, MP increased the expression level of CD144 in non-inflammatory and inflammatory conditions. As in macrophages, MP also induced a pro-inflammatory response in HUVEC. MP upregulated gene expression of pro-inflammatory cytokines IL-6 in inflammatory HUVEC and IL-8 in non-inflammatory and inflammatory HUVEC. MP downregulated gene expression of endothelin under both conditions (Figure 6C).

## Discussion

In this study, we investigated the mechanisms of MP uptake and its effect on macrophage and HUVEC phenotype. Our findings revealed that MSC-derived MP enter macrophages and HUVEC through various mechanisms, involving receptor-mediated endocytosis, macropinocytosis, and phagocytosis, and modulate macrophage and HUVEC function. Identification of mechanisms involved in MP internalization under different conditions allows specific modulation of MP delivery to target cells.

MSC-derived MP therapy has several advantages over MSC themselves. MP contain the membrane-bound proteins of MSC and the expression of these proteins on MP is not modified by the environment after infusion as in living cells. Since MP are not a living cellular product, there is no risk of transformation after administration. Because of their small size, MP are better capable of crossing the pulmonary barrier than MSC. Unlike the collection of naturally occurring extracellular vesicles, which are a mixture of vesicles budding off from the cell membrane and endoplasmic reticulum, MP generation is a simple, low-cost, and scalable process. The MP production process offers a number of possibilities to modify their activity, which would be more complex for extracellular vesicles. Firstly, as the protein make-up of MP mirrors that of their mother MSC, treatment of MSC with for instance pro-inflammatory cytokines to induce the expression of membrane bound proteins with anti-inflammatory function or lentiviral transfection of MSC to induce the expression of a particular protein of interest would result in the incorporation of such proteins in MP generated from these MSC. Secondly, the closure of membrane fragments into circular membrane particles allows the enclosure of drugs of interest, which will be delivered to target cells upon MP uptake. In this way target cells can be treated with MP and simultaneously with a drug.

The focus of the present study was on mechanisms of MP uptake and internalization by two cell types with barrier and immune patrolling functions, namely, endothelial cells (represented by HUVEC) and macrophages (represented by THP-1 macrophages). Cellular and extracellular environments, such as temperature, exposure time, inflammatory environment, and the available uptake machinery in target cells can govern the bio-distribution of MP (19, 24). We established that macrophages and HUVEC uptake MP in a dose-, time, and temperature-dependent manner and internalized MP into the cytoplasm. In our previous studies, we showed that MP do not physically interact with T cells, but interact with monocytes and HUVEC by binding to the plasma membrane through fusion and internalization, respectively (16) (unpublished data). This discrepancy might be explained by the fact that MP-cell interaction is strictly dependent on cell contact, but T cell activation requires soluble factors. Therefore, the interaction of MP with the plasma membrane of macrophages and HUVEC supports the idea that MP can be a natural delivery vehicle for macrophage and endothelial cell targeting drugs. These results need to be confirmed in primary cells as it is possible that the cell lines used in the present paper generate different results than primary cells.

In the present study, we choose to explore the mechanisms of MP uptake by the use of a number of inhibitors at concentrations that did not induce cell death within 6 h. We cannot rule out the possibility that longer incubations would lead to cell death, or that mild cytotoxicity, not resulting in cell death within 6 h, did occur. However the involvement of some key pathways in MP uptake was already observed at concentrations far below necrosis-inducing doses. Six different mechanisms of endocytosis were addressed in this study: HSPG-, dynamin-, clathrin-, caveolin-mediated endocytosis, phagocytosis, and macropinocytosis. Our data revealed that MP bind to cells *via* cell-surface receptors and are later internalized through HSPG-, dynamin-, and clathrin-mediated endocytosis. HUVEC internalized MP *via* clathrin-coated pits more efficiently than macrophages, suggesting that there are differences in the dominant uptake pathways between different cell types (endothelial cells express more clathrin on their cell membrane). In addition, we found that MP uptake is a caveolin-independent process. Several studies report that caveolin-independent, but dynamin-dependent endocytosis is involved in the formation of non-coated vesicles in the plasma membrane (21, 24, 27, 28). In cells without caveolin, the same dynamin-dependent pathway can functionally replace caveolar endocytosis (29). Moreover, studies suggest that the internalization of particles is limited to particles smaller than the size of caveolin (about 50–100 nm) (30, 31). However, inhibition of caveolin pathway slightly increased uptake of MP by HUVEC. We hypothesize that alterations in the caveolin pathway can stimulate other uptake mechanisms as a result of endocytic compensation.

We showed that MP internalization by macrophages and HUVEC also depends on the actin cytoskeleton and PI3K through phagocytosis and macropinocytosis process. Macrophages internalized MP *via* actin-mediated mechanisms more efficiently than HUVEC, indicating that MP are preferentially internalized by phagocytes cells (24). Moreover, combination treatment that interferes with HSPG-, dynamin, and actin-mediated mechanism almost completely blocked MP internalization, suggesting that receptor-mediated endocytosis, and phagocytosis are processes that independently contribute to MP uptake.

Our findings demonstrated that anti-inflammatory M2 macrophages take up more efficiently MP than pro-inflammatory M1 macrophages. Tumor-associated macrophages exhibit the tumor-promoting M2 phenotype rather than the tumor-suppressing M1 phenotype (32). In the early stage of tumor development, pro-inflammatory stimuli recruit monocytes and polarize them into M1 macrophages (33), which inhibit cancer progression and angiogenesis (34–36). However, in later stage, tumor cells induce the differentiation of M1 macrophages into M2 and in this way escape the immune system and support tumor progression (37, 38). The efficient uptake of MP by M2 macrophages accompanied by increase production of CCL22 and CD209, which recruit regulatory T cells and dendritic cells into cancer tissue (39, 40), enhanced the M2 immune activity and can be used to target macrophages involved in cancer progression. Such activity of MP could be further enhanced by loading MP with anti-cancer drugs. Despite the low uptake by M1 macrophages, MP regulated inflammation, and induced the expression of tumor inhibitory cytokines IL1-β and TNFα (34–36), indicating that M1 macrophages may be a potential target for MP therapy in inflammatory diseases and cancer.

Moreover, we demonstrated that MP promoted both pro- and anti-inflammatory effects on macrophages and HUVEC under different stimuli. We speculate that MP contain both pro- and anti-inflammatory proteins from MSC and induce gene expression changes in target cells and, subsequently, support the dynamic immunomodulatory activities of cell repair and regeneration. In one of our previous studies, we detected intact mRNA for VEGF, IL-8, and CD90 from the MSC on MP samples (unpublished data). In the other study, we found CD90 from MSC was upregulated on monocytes after interaction with MP (16). However, further studies are required to clarify the MP components.

Taken together, this study broadens our knowledge on the molecular pathways involved in the uptake of MP by macrophages and HUVEC and on the effects of MP uptake on cellular function. This knowledge can lead to the design of MP with target cell specificity under particular inflammatory conditions. MP thereby become a potential novel tool to modulate inflammatory responses in immune and degenerative diseases and cancer.

## Data Availability Statement

The raw data supporting the conclusions of this article will be made available by the authors, without undue reservation.

## Author Contributions

FC planned the research, performed experiments, and wrote the manuscript. SK performed experiments. MO, AM, and EL planned the research and reviewed the manuscript. CB and MR reviewed the manuscript. MH planned the research and wrote the manuscript. All authors contributed to the article and approved the submitted version.

## Conflict of Interest

MO and EL were employed by Takeda Madrid. Erasmus MC filed a patent on MSC derived MP (PCT/NL2017/050334). The remaining authors declare that the research was conducted in the absence of any commercial or financial relationships that could be construed as a potential conflict of interest.

## Supplementary Material

Supplementary Figure 1 Characterization of MP and PKH-MP. **(A)** Nanoparticle tracking analysis (NTA) profile of MP and PKH-MP. **(B)** The size distribution of MP and PKH-MP.

Supplementary Figure 2 Macrophages and HUVEC viability after treating with selective inhibitors. Cells were preincubated for 30 min in complete medium containing Heparin (0.1–100 μg/ml), Dynasore (20–160 μM), Chlorpromazine (1–50 μM), Nystatin (5–40 μg/ml), Cytochalasin D (0.25–2 μM), or Wortmannin (0.1–10 μM). PBS and 0.1% DMSO were used as a control. Cell viability was measured by flow cytometer after 6 h using 7-AAD. Percentage of macrophage **(A)** and HUVEC **(B)** viability. ^*^*P* < *0.05* and ^***^*P* < *0.001 vs*. vehicle.

Supplementary Figure 3 Macrophage profile under different culture conditions. Macrophages were primed with 20 ng/ml IFNγ and 100 ng/ml LPS (inflammatory condition), or 20 ng/ml IL-4 and 20 ng/ml IL-13 (anti-inflammatory condition). **(A)** Photomicrographs showing characteristic morphologies of macrophages exposed to different culture conditions for 24 h (Magnification 100x). **(B)** mRNA expression of macrophages by real-time RT-PCR for CXCL10, CCR7, IL-1β, IL-10, TNFα, TGFβ, CCL22, and CD209 after treated with IFNγ + LPS or IL-4 + IL-13 for 72 h. Data are presented as mean ± SD from 6 experiments. ^*^*P* < *0.05*, ^**^*P* < *0.01*, and ^***^*P* < *0.001 vs*. medium control.

Supplementary Figure 4 HUVEC profile under different culture conditions. HUVEC were primed with single or combined doses of 10 ng/ml TNFα and 50 ng/ml IFNγ and assessed by flow cytometer and real-time RT-PCR. **(A)** Photographs showing characteristic morphologies of HUVEC exposed to different culture conditions for 24 h (Magnification 100×). **(B)** HUVEC surface levels of HLA-I, HLA-II, CD40, and CD144 and **(C)** mRNA expression of HUVEC for IL-6, IL-8, and endothelin in the presence of single or combined doses of TNFα and IFNγ after 72 h. Data are presented as mean ± SD from 6 experiments. ^*^*P* < *0.05*, ^**^*P* < *0.01*, and ^***^*P* < *0.001 vs*. medium control.
