# Supplementary figures and images for: Mesenchymal Stromal Cell Derived Membrane Particles Are Internalized by Macrophages and Endothelial Cells Through Receptor-Mediated Endocytosis and Phagocytosis
Source: Front Immunol. 2021 Mar 15;12:651109. doi: 10.3389/fimmu.2021.651109 (PMC8005704; doi:10.3389/fimmu.2021.651109)

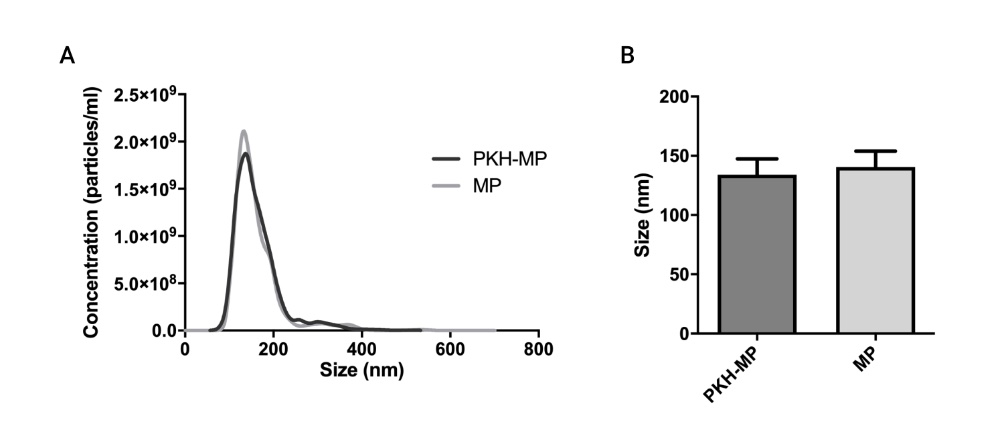

Supplement: Supplementary Figure 1 — Characterization of MP and PKH-MP. (A) Nanoparticle tracking analysis (NTA) profile of MP and PKH-MP. (B) The size distribution of MP and PKH-MP. [file Image_1.jpg]

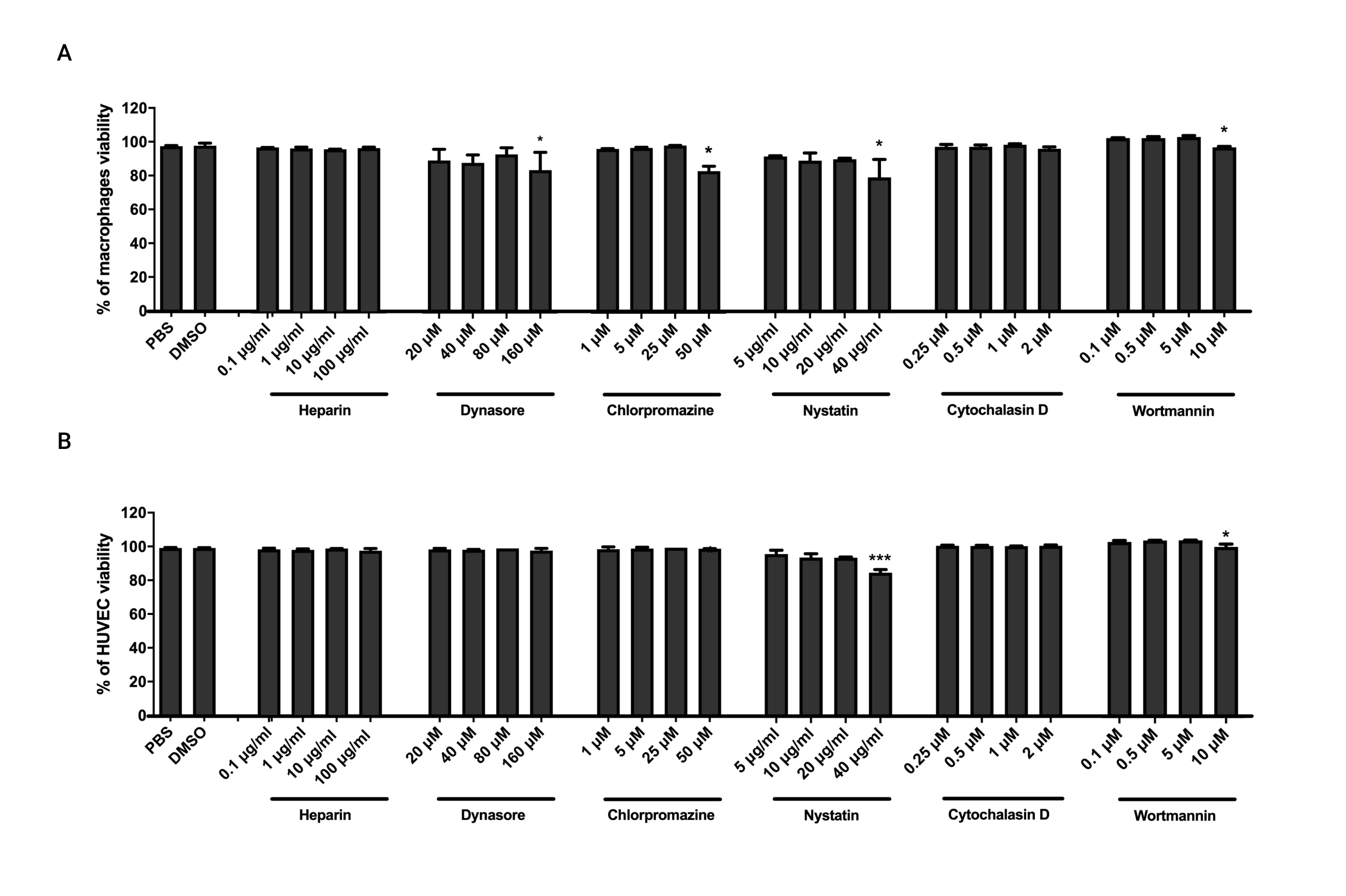

Supplement: Supplementary Figure 2 — Macrophages and HUVEC viability after treating with selective inhibitors. Cells were preincubated for 30 min in complete medium containing Heparin (0.1–100 μg/ml), Dynasore (20–160 μM), Chlorpromazine (1–50 μM), Nystatin (5–40 μg/ml), Cytochalasin D (0.25–2 μM), or Wortmannin (0.1–10 μM). PBS and 0.1% DMSO were used as a control. Cell viability was measured by flow cytometer after 6 h using 7-AAD. Percentage of macrophage (A) and HUVEC (B) viability. *P < 0.05 and ***P < 0.001 vs. vehicle. [file Image_2.jpg]

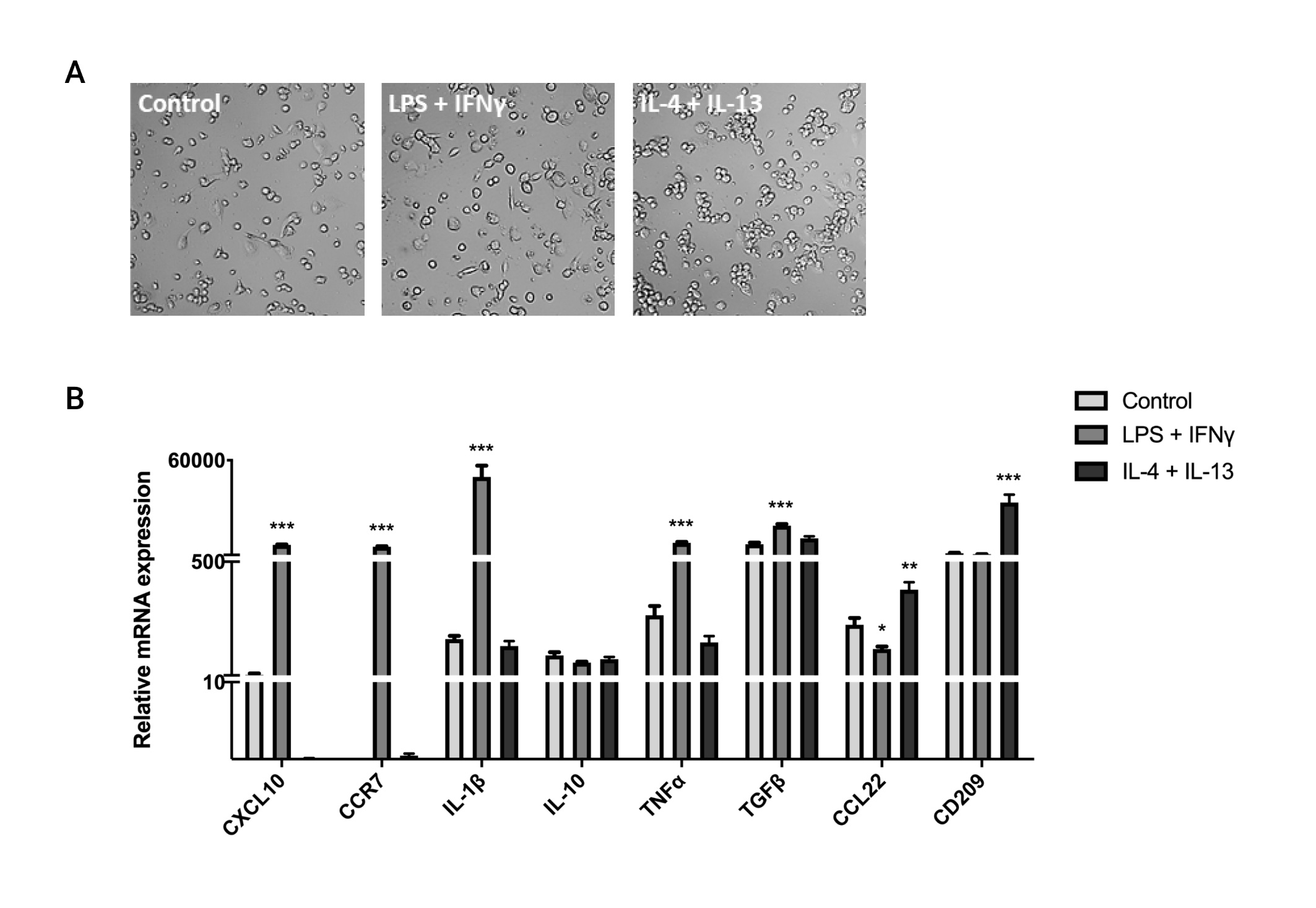

Supplement: Supplementary Figure 3 — Macrophage profile under different culture conditions. Macrophages were primed with 20 ng/ml IFNγ and 100 ng/ml LPS (inflammatory condition), or 20 ng/ml IL-4 and 20 ng/ml IL-13 (anti-inflammatory condition). (A) Photomicrographs showing characteristic morphologies of macrophages exposed to different culture conditions for 24 h (Magnification 100x). (B) mRNA expression of macrophages by real-time RT-PCR for CXCL10, CCR7, IL-1β, IL-10, TNFα, TGFβ, CCL22, and CD209 after treated with IFNγ + LPS or IL-4 + IL-13 for 72 h. Data are presented as mean ± SD from 6 experiments. *P < 0.05, **P < 0.01, and ***P < 0.001 vs. medium control. [file Image_3.jpg]

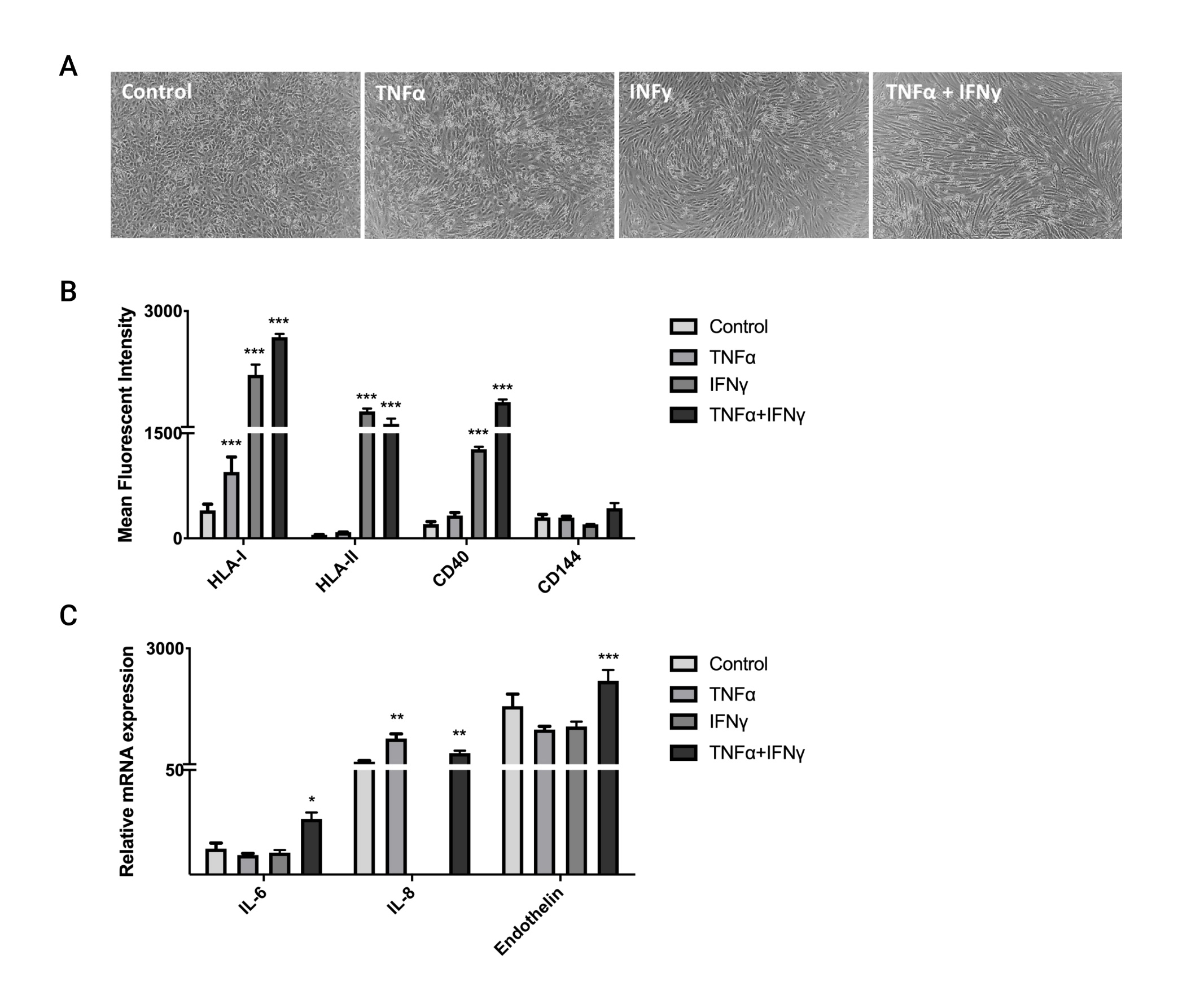

Supplement: Supplementary Figure 4 — HUVEC profile under different culture conditions. HUVEC were primed with single or combined doses of 10 ng/ml TNFα and 50 ng/ml IFNγ and assessed by flow cytometer and real-time RT-PCR. (A) Photographs showing characteristic morphologies of HUVEC exposed to different culture conditions for 24 h (Magnification 100×). (B) HUVEC surface levels of HLA-I, HLA-II, CD40, and CD144 and (C) mRNA expression of HUVEC for IL-6, IL-8, and endothelin in the presence of single or combined doses of TNFα and IFNγ after 72 h. Data are presented as mean ± SD from 6 experiments. *P < 0.05, **P < 0.01, and ***P < 0.001 vs. medium control. [file Image_4.jpg]
